# Unveiling the functional landscape of A-to-I RNA editing associated with Marek’s disease in chickens

**DOI:** 10.1371/journal.ppat.1014743

**Published:** 2026-11-05

**Authors:** Qunhao Niu, Yi Ding, Longbin Yang, John R. Dunn, Huanmin Zhang, Keji Zhao, Jiuzhou Song

**Affiliations:** 1 Department of Animal and Avian Science, University of Maryland, College Park, Maryland, United States of America; 2 Brain Health, Allen Institute for Brain Science, Seattle, Washington, United States of America; 3 U.S. Department of Agriculture, Agricultural Research Service, U.S. National Poultry Research Center, Athens, Georgia, United States of America; 4 Laboratory of Epigenome Biology, Systems Biology Center, National Heart, Lung and Blood Institute, NIH, Bethesda, Maryland, United States of America; Oklahoma State University College of Osteopathic Medicine, UNITED STATES OF AMERICA

## Abstract

Genetic improvement of disease resistance against viral infections represents a longstanding and formidable challenge in animal breeding. The antiviral innate immune response is highly regulated at the RNA level, especially through RNA modifications. Adenosine-to-Inosine (A-to-I) RNA editing modifies the mRNA sequence. Growing evidence shows that this type of RNA editing plays a key role in mediating cellular responses to viral infections and disease resistance. However, the role of RNA editing in cellular response to MD remains unclear. In this study, we examined A-to-I editing in CD4^+^ T cells during Marek’s disease virus (MDV) infection. By comparing MD-resistant and susceptible chicken lines, we identified 642 A-to-I RNA editing events in CD4^+^ T cells, most of which were located within repeat elements, particularly in the CR1 family. We observed differences in RNA editing levels and counts between the two chicken lines with different infection statuses, indicating distinct editing patterns across genetic backgrounds and infection states. Additionally, *ADAR* (adenosine deaminases acting on RNA) expression was correlated with RNA editing frequency. We identified 21 RNA editing sites (RESs) associated with gene expression and 42, 14, and 38 RESs with predicted effects on amino acid sequences, alternative splicing, and miRNA-target binding, respectively. A comparative analysis revealed 131 group-specific RESs and 21 RESs shared across groups. Functional enrichment analysis of genes associated with group-specific RESs highlighted several terms related to MD resistance, including the MHC class I protein complex in non-infected MD-resistant birds. Significantly, we identified two L6_3_-specific candidate RNA editing sites, chr6:26,600,122 in *MXI1* and chr16:1,716,632 in *MHCY57*, which were predicted to affect gga-miR-1759-3p binding and amino acid sequence, respectively. Our findings reveal the landscape of A-to-I RNA editing in CD4 ⁺ T cells during MDV infection and provide new insights into its potential association with immune regulation and MD resistance.

## Author summary

Marek’s disease (MD) is a contagious viral disease of chickens that causes immune suppression, tumors, and economic losses to the poultry industry. Lines 6_3_ and 7_2_ are well-established chicken models for MD resistance and susceptibility, respectively, but the molecular basis of this difference remains unclear. We investigated whether RNA editing, a process that changes RNA after it is produced, is associated with differences in host responses between MD-resistant and MD-susceptible chickens. By examining splenic CD4⁺ T cells from MDV-infected and uninfected chickens of the resistant and susceptible lines, we identified hundreds of RNA editing events. These events were associated with differences in gene expression or were predicted to affect protein sequences, RNA processing, and interactions with small regulatory RNAs. We also identified two editing events in resistant chickens that may influence genes involved in immune regulation and tumor control. Our study highlights candidate RNA editing events associated with differential host responses to MDV infection. These findings improve our understanding of disease resistance and may inform future disease-resistant breeding and control strategies.

## Introduction

Recent discoveries have documented how RNA modifications play key roles in cellular surveillance of nucleic acids and in controlling gene expression in response to viral infection [1]. These modifications have emerged as being essential for a functional antiviral response and maintaining cellular homeostasis [2]. Adenosine-to-inosine (A-to-I) RNA editing is a post-transcriptional modification that regulates the innate immune response in host cells during viral infections [3]. Marek’s disease (MD) is a highly contagious, neuropathic, and lymphomatous disease of chickens caused by Marek’s disease virus (MDV), an oncogenic alphaherpesvirus [4]. MDV infection progresses through cytolytic, latent, and T-cell transformation phases, leading to widespread tumors and high mortality. The disease severely impairs meat quality, feed efficiency, egg production, immune function and poultry welfare [5]. Despite extensive vaccination, MD outbreaks still occur worldwide, causing substantial economic losses and posing a persistent threat to the global poultry industry.

Two highly inbred White Leghorn chicken lines, 6_3_ and 7_2_ lines, which were developed from the same ancestor, have been selectively bred and serve as well-established experimental models for MD research, with line 6_3_ (L6_3_) exhibiting resistance and line 7_2_ (L7_2_) showing susceptibility to MDV-induced tumors [6]. Extensive studies have been conducted to investigate the genetic basis underlying the divergent resistance phenotypes of these two chicken lines from multiple biological perspectives, including selective sweeps [7], copy number variations [8], gene expression profiling [9], and epigenomic regulation [10–13]. These studies have advanced our understanding of MDV-associated immune responses, highlighting the intricate interplay between genomic, transcriptomic, and epigenomic regulatory mechanisms in poultry. As a post-transcriptional regulatory mechanism, A-to-I RNA editing may provide further insights into the molecular basis of differences in MD resistance between these two lines.

A-to-I RNA editing, mediated by adenosine deaminase acting on RNA (ADAR) enzymes, is the most abundant form of RNA editing in vertebrates and catalyzes the conversion of adenosine to inosine within double-stranded RNA [14]. Previous studies have shown that A-to-I RNA editing is associated with alternative splicing, miRNA-target binding, mRNA isoform diversity, protein recoding, and gene silencing, contributing to transcriptome diversity and driving adaptive evolution and phenotypic variation [15–17]. Extensive A-to-I RNA editing events have been identified across multiple tissues, providing valuable insights into evolutionary conservation, tissue-specific regulatory patterns, and phenotypic variation in farm animals, including chickens [18], cattle [19], goats [20], and pigs [21]. CD4^+^ T cells are the primary target of MDV, which infects and transforms them into cancerous lymphomas, resulting in multiple visceral tumors and immunosuppression in chickens [22,23]. Previous studies have suggested that A-to-I RNA editing is also an essential regulator of innate immunity and cell death [24,25]. For example, ADAR1-mediated A-to-I RNA editing regulates cytokine secretion and differentiation in CD4⁺ T cells, and its dysregulation can impair thymic negative selection, leading to autoimmune diseases [26], promote interferon signaling and T-cell survival, and has been implicated in maintaining antiviral immunity and immune homeostasis after viral infection [27]. However, RNA editing in regulating immune responses and disease resistance to MDV infection in chickens remains largely unexplored.

Given the established roles of ADAR-mediated A-to-I RNA editing in antiviral responses and immune regulation, we hypothesized that MD-resistant and -susceptible chickens may exhibit distinct RNA editing profiles in CD4^+^ T cells following MDV infection, with potential consequences for immune-related gene regulation. In this study, we systematically characterized A-to-I RNA editing events in CD4^+^ T cells from MDV-infected and uninfected chickens of the two genetic lines. We examined the associations between *ADAR*/*ADARB1* expression and RNA editing events, evaluated the relationship between RNA editing and gene expression, and assessed the predicted effects of editing on protein-coding sequences, alternative splicing, and miRNA-target interactions. We further compared editing patterns between the resistant and susceptible lines to identify candidate sites associated with differential host responses to MDV infection. Together, these analyses provide new insights into the potential involvement of A-to-I RNA editing in immune regulation associated with MD resistance.

## Results

### Genome-wide identification of A-to-I RNA editing sites in chicken CD4^+^ T cells

CD4^+^ T cell lymphoma induced by MDV may promote the development of multiple visceral neoplasms and immunosuppressive states in MD-infected chickens. Here, we generated transcriptomic datasets for CD4^+^ T cells from two chicken lines at 10 days post-MDV infection (DPI), along with control samples for comparative analysis (Fig 1A). We detected A-to-I RNA editing sites (RESs) using REDItools3. We obtained high-confidence RESs after multi-step filtering, including discarding SNPs in the European Variation Archive and our own population (detailed filters are provided in the Methods). Finally, we detected a total of 642 A-to-I editing events across 12 CD4^+^ T cells samples, with at least 3 coverage reads. These sites were located at 187 non-redundant positions across 33 autosomes in chicken (S1 Table). Among the identified RNA editing sites, 148 unique RESs, represented by 476 editing events, were supported by more than 10 total reads, accounting for 79.14% of all unique RESs (S1A, S1B Fig). The editing level for each site was calculated as the proportion of edited reads to total reads. We observed that the editing levels of most RESs ranged from 0 to 0.25 (S2A Fig). We found that most A-to-I editing events (57.8%) were detected in only two samples (Fig 1B). The highest quantity of RESs was detected in the MDV-infected group of Line 7_2_. Moreover, we observed significant differences between infected and non-infected groups across the two chicken lines. Specifically, L7_2_ exhibited a higher number of RESs in the MDV-infected group than the control group, whereas L6_3_ displayed an opposing trend (Fig 1C). However, editing levels did not differ significantly between the MDV-infected and control groups in either chicken line. A significant difference in editing levels was observed between MD-susceptible and MD-resistant lines, with MD-resistant lines showing higher editing levels, suggesting that this pattern may be associated with line-specific differences in MD resistance and susceptibility (Fig 1D).

**Fig 1:**
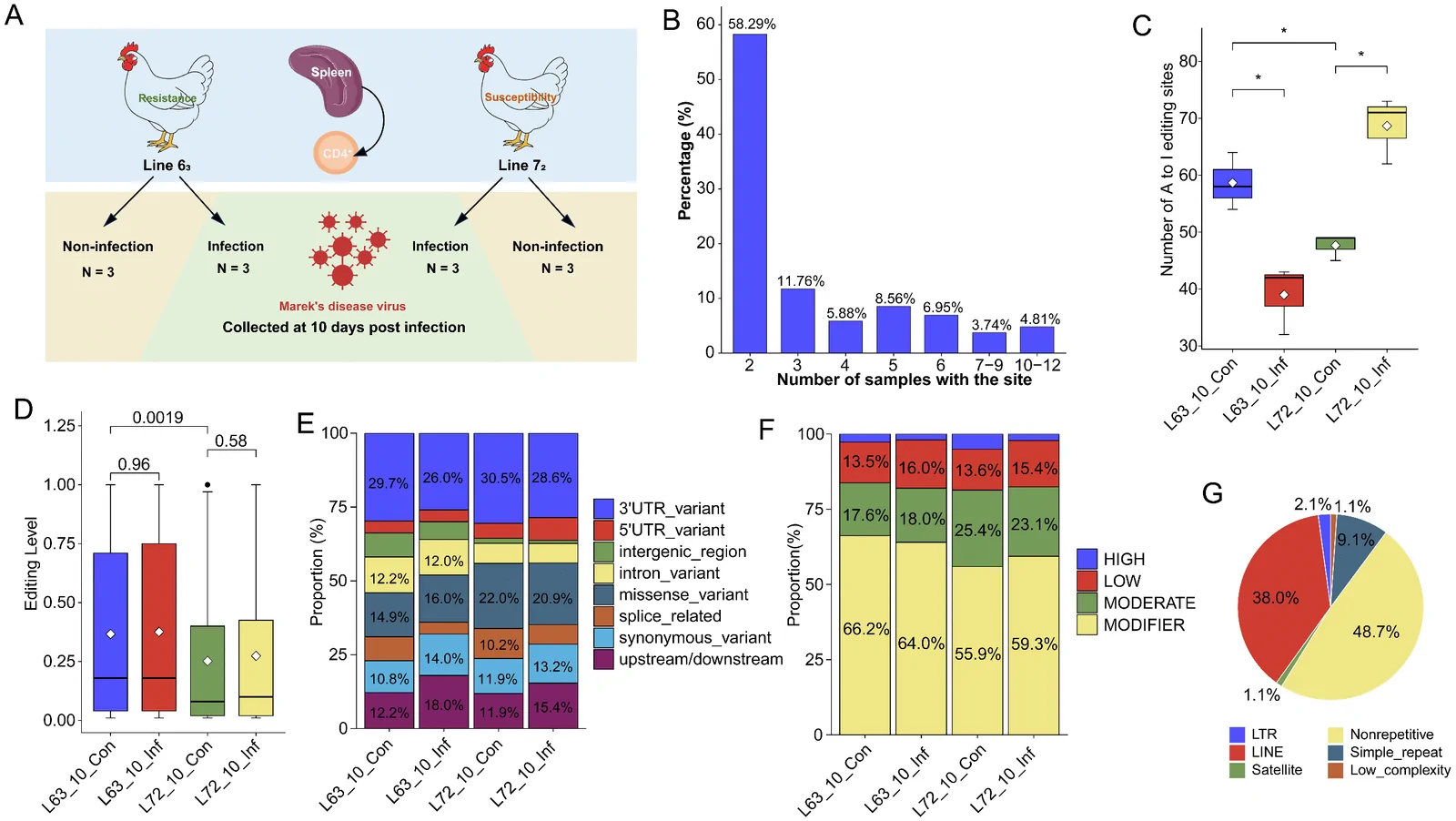
Landscape of A-to-I RNA editing in CD4^+^ T cells from MD-resistant and MD-susceptible chickens with different infection status. **A.** The experimental design for the identification of RNA editing in chicken CD4^+^ cells. CD4^+^ T cells were isolated from spleens with different Marek’s disease virus (MDV) infection status for two chicken lines at DPI 10. **B.** The distributed percentage of A-to-I editing sites (RESs) across individuals. **C.** Boxplot comparing the number of detected RESs per sample across the four groups. (L63_Con, L6_3__Inf, L7_2__Con, and L7_2__Inf). *, *P* ≤ 0.05 (Wilcoxon rank-sum tests). **D.** Boxplot comparing RNA editing levels across the four groups. *P-*values are marked in the plot (Wilcoxon rank-sum tests). **E.** Genomic annotation for RESs by SnpEff across four groups. **F.** Predicted impact categories of RESs annotated by SnpEff, including HIGH, MODERATE, LOW, and MODIFIER. **G.** Distribution of RESs across different chicken repeat elements.

To explore the functional feature of A-to-I RESs in chickens, we performed annotation of 187 unique RESs. Of these, 145 RESs mapped to genes; nearly all resided in protein-coding genes, while only 5 were localized to lncRNAs and 1 to miRNAs. Across all four groups, we found that A-to-I RESs were predominantly annotated to the 3’UTR region (28.70%), followed by missense variants (18.40%) and upstream/downstream variants (14.40%) (Fig 1E), and most of them were predicted to have indirect regulatory effects or moderate impacts on protein structure (Fig 1F). Moreover, we found that over half of the A-to-I RESs were localized to repeat elements within the chicken genome, including long terminal repeats (LTRs), long interspersed nuclear elements (LINEs), and simple repeats. Notably, RESs residing in LINEs accounted for 38% of all identified RESs, with the vast majority annotated as members of the CR1 family (Fig 1G).

### The relationship between A-to-I editing sites and gene expression of *ADAR* and *ADARB1*

A-to-I RESs on the chicken genome are mediated by the *ADAR* enzymes such as ADAR and ADARB1. In this study, we observed gene expression of *ADAR* and *ADARB1*. For *ADARB1*, we observed differential expression across three comparison groups spanning distinct MDV infection statuses and chicken lines, whereas *ADAR* gene expression showed a significant difference only between MDV-infected and control samples in L7_2_ (Fig 2A). We explored the potential relationship between *ADAR* gene expression and RES number and editing level. We found only a marginally significant positive correlation between *ADAR* expression and the number of RESs (Fig 2B, 2C, S2B, S2C Fig). Furthermore, we performed Wilcoxon rank-sum tests to identify RESs whose presence was associated with *ADAR* or *ADARB1* expression in CD4⁺ T cells. We identified 8 and 18 RESs associated with *ADAR* and *ADARB1* expression, respectively. The expression of *ADAR* and *ADARB1* exhibited a significant difference between samples harboring these RESs and those devoid of the corresponding RESs (S2 Table, S2D, S2E Fig). For instance, a RES at chr4:1,848,128, whose presence may be associated with *ADAR* expression, was located in *RPS4Y1* and annotated as a missense variant, potentially affecting the involvement of *RPS4Y1* in virus-host protein interactions [28] (Fig 2D)*.* A RES at chr3:55,866,463 was identified in *SGK1*, a key regulator of immune-cell function, and its presence was associated with *ADAR* expression [29] (Fig 2D). In addition, we detected a RES at chr16:1,716,632, whose presence was associated with *ADARB1* expression, located in the genic region of *MHCY57*, a gene within the MHC-Y haplotype associated with disease resistance in chickens (Fig 2E). Another RES (chr1:182,466,974) in *BIRC2*, a gene previously implicated in MD resistance of Line 6 chickens, was annotated as a predicted high-impact splice-acceptor variant and was also associated with *ADARB1* expression [30] (S2E Fig).

**Fig 2:**
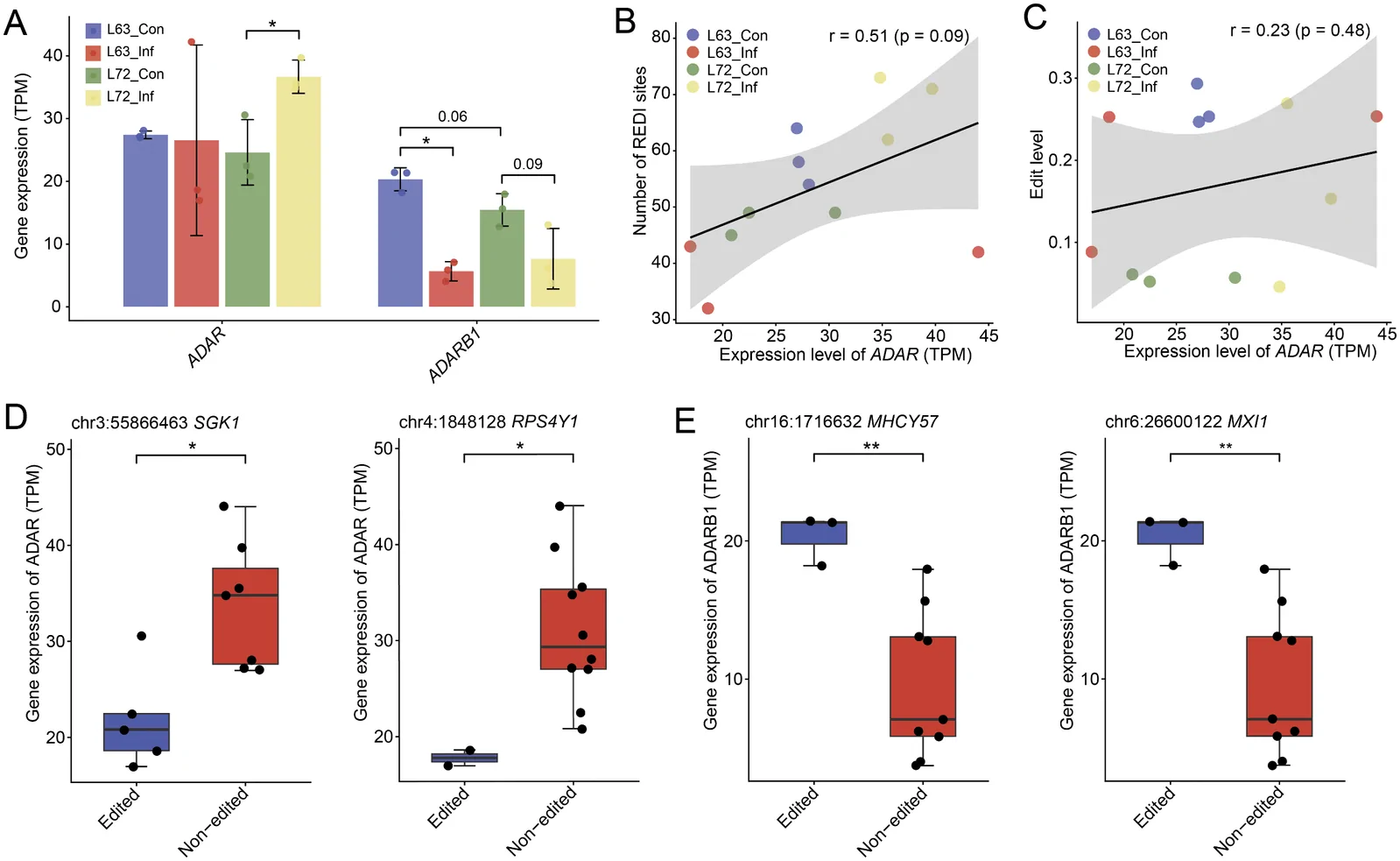
Relationship between *ADAR/ADARB1* expression and A-to-I RNA editing patterns in chicken CD4^+^ T cells for Marek’s disease. **A.** Bar plot shows the comparison of gene expression for *ADAR* and *ADARB1* across the four groups (L63_Con, L63_Inf, L72_Con, and L72_Inf). **B.** Correlation between *ADAR* expression and the number of RESs detected per sample. Each point represents one biological replicate. The solid line indicates linear regression, with the shaded area denoting the 95% confidence interval. **C.** Correlation between *ADAR* expression and RNA editing levels. Editing level was calculated as the ratio of edited reads to total reads. **D.** Comparison of *ADAR* expression between samples with and without RNA editing at two representative *ADAR*-associated RESs located in *SGK1* (chr3:55,866,463) and *RPS4Y1* (chr4:1848128). **E.** Comparison of *ADARB1* expression between samples with and without RNA editing at two representative *ADARB1*-associated RESs located in *MHCY57* (chr16:1,716,632) and *MXI1* (chr6:26,600,122). Statistical significance was assessed using Wilcoxon rank-sum tests (*P < 0.05, **P < 0.01).

### Gene expression associations and predicted consequences of A-to-I RNA editing in CD4^+^ T cells

To investigate the potential consequences of RESs in chicken MD, we performed a series of analyses to explore their potential regulatory roles in gene expression. For gene expression, we detected 21 RESs associated with 19 unique genes, and these genes exhibited nominally significant differences in expression levels between edited and unedited samples (Wilcoxon rank-sum test, *P* <= 0.05) (Fig 3A, S3 Table). We found that samples with RESs tended to show higher expression of the corresponding genes. For example, samples with the editing site (chr11:18,171,757) located in the 3’UTR had higher gene expression of *LOC107057295.* Notably, 10 of the 21 RESs were classified as *ADAR*- or *ADARB1*-associated sites. Based on SnpEff annotations of RESs, we identified a total of 41 missense sites and 1 start codon gain site that may result in amino acid alterations. Meanwhile, 14 sites were predicted to be associated with alternative splicing events, of which 8 splicing-related sites were located in intronic regions, and 4 were additionally predicted to have missense functions (S4 Table). We further evaluated the potential effect of 49 RESs located in the 3’UTR region on miRNA-target binding. A total of 117 predicted miRNA-targets were identified to be affected by 38 editing events, including 64 edited gains and 53 edited losses, impacting 32 mRNAs (S3A Fig, S5 Table). Notably, the presence of RESs was predicted to alter miRNA-target binding of gga-miR-1759-3p with 3’UTRs of six target genes, including *GPX4*, *MXI1*, *TMSB15B*, *MATN*, ENSGALG00010030065, and ENSGALG00010019361 (Fig 3B). Similarly, we found the editing event (chr17:6695396) was predicted to affect the binding of seven miRNAs to the 3’UTR region of *NUP214* (Fig 3B). Based on GO and KEGG analyses, we found that genes with miRNA targets affected by RESs were enriched in arachidonic acid metabolism and the notch signaling pathway. In summary, we identified 21, 42, 14, and 38 RESs associated with gene expression, amino acid sequences, alternative splicing, and miRNA-target binding, respectively. Notably, 15 of these RESs were predicted to have at least two distinct functional consequences (Fig 3C and S3B Fig). For example, an *ADARB1*-associated RES (chr5:37,730,588) was associated with *FOS* expression and was predicted to alter the amino acid sequence. Another *ADARB1*-associated RES (chr28:4,187,743) was relevant to *GDF3* expression and was predicted to alter miRNA binding within the 3’ UTR, including interactions with gga-miR-1604, gga-miR-29a-3p, gga-miR-6570-5p, and gga-miR-9-4-3p (Fig 3C, S5 Table). Furthermore, we extracted the 10 bp flanking region of 187 A-to-I RESs to examine their base preference. We found that G is enriched at the 1 bp downstream of editing events, but observed no preference for any base at 1 bp upstream (Fig 3D). A total of 32 RESs are located in 97 coding regions for 47 genes whose codons present a preference for G or C bases (Fig 3D). The occurrence of RES may alter amino acid types and preferences, resulting in a higher proportion of glycine, arginine, and alanine (Fig 3D). Collectively, our results suggest an association between A-to-I RNA editing and gene expression in CD4⁺ T cells and multiple predicted regulatory consequences including alternative splicing, miRNA-target interactions, and amino acid sequences.

**Fig 3:**
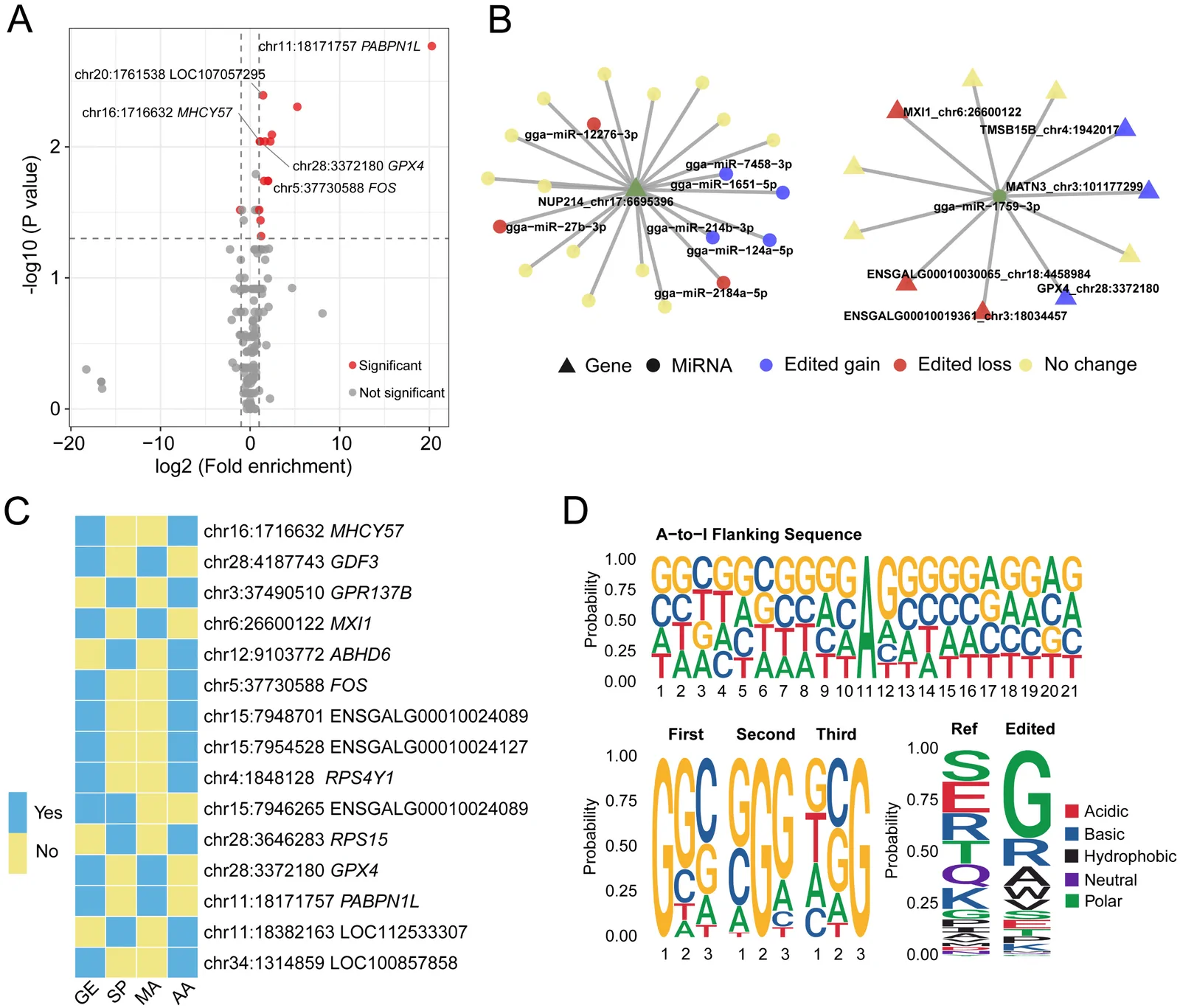
Gene expression associations and predicted effects of A-to-I RNA editing in CD4^+^ T cells. **A.** Volcano plot was generated to display differential gene expression between edited and non-edited samples at target RESs using the Wilcoxon rank-sum test. Each point represents one RES-gene association across the 12 samples. For each RES, expression of the corresponding gene was compared between samples in which the editing site was detected and those in which it was not detected using the Wilcoxon rank-sum test. The x-axis indicates the log2 fold change in gene expression between RES-positive and RES-negative samples, and the y-axis indicates the −log10 nominal P value. Red points indicate nominal associations based on unadjusted *P* < 0.05, whereas gray points indicate non-significant associations. Selected RESs associated with immunity regulation are labeled with the genomic coordinate of the RES and the corresponding gene symbol. **B.** Two examples of miRNA-target binding predicted by RESs. The left panel shows a RES (chr17:6,695,396) located within *NUP214* that was predicted to result in the loss of three miRNA binding events and the gain of four new binding events after RNA editing. The right panel illustrates that predicted miRNA binding of gga-miR-1759-3p to the 3’UTRs of six genes can be affected by RESs. **C.** Heatmap summarizing selected RESs represented in at least two analysis categories: nominal gene expression associations (GE) and computationally predicted effects on alternative splicing (SP), miRNA-target interactions (MA), and amino acid sequences (AA). **D.** Comparison of base preferences in the flanking regions of RESs, codon sequences, and the encoded amino acids. The height of each letter represents its relative frequency at each position.

### Differential and group-specific A-to-I editing events for Marek’s disease

To detect RESs and associated genes potentially related to MD resistance, we first identified 849, 576, and 607 significantly differentially expressed genes (DEGs) in the L6_3_-infection vs. L6_3_-control, L7_2_-infection vs. L7_2_-control, and L6_3_-control vs. L7_2_-control comparisons, respectively (S6 Table). We then employed Fisher’s exact test on pooled RNA reads for both reference and edited bases, yielding 4, 5, and 2 differential RESs across the same three groups (S7 Table). In the L6_3_-infected vs. L6_3_-control comparison, an editing site located in the 3’ UTR of *HMGB3* (chr4:17,601,347) showed an increased editing level in infected individuals and was strongly negatively correlated with *HMGB3* expression. Notably, *HMGB3* encodes a chromatin-associated protein expressed in hematopoietic stem and progenitor cells, where it contributes to the regulation of stem-cell self-renewal and lymphoid and myeloid differentiation and has also been implicated in innate immune regulation [31,32]. Conversely, a synonymous site (chr19:7,071,499) with a decreasing editing level in infected individuals shows a positive correlation with gene expression of *UBB* (Fig 4A, 4B). Moreover, several important genes were also pinpointed by differential RESs, such as the splicing-related intron site (chr1:182,466,974) of *BIRC2* and the 3’UTR site (chr25:1,196,928) of *SLAMF1* for higher editing levels in the L7_2_-infected group. Integrating DEG results, we identified four differential RESs within two DEGs, including ENSGALG00010024056, ENSGALG00010024069, across three groups (Fig 4C). A missense RES (chr15:7,943,040) had a higher editing level in control samples than infected samples in both chicken lines; its corresponding gene (ENSGALG00010024056) exhibited a higher gene expression in the L6_3_-infection group but a lower expression in the L7_2_-control group in their respective comparisons. This gene was also identified as a candidate for line difference by another missense site (chr15:7,947,337) with a higher editing level and gene expression in L7_2_ (Fig 4D).

**Fig 4:**
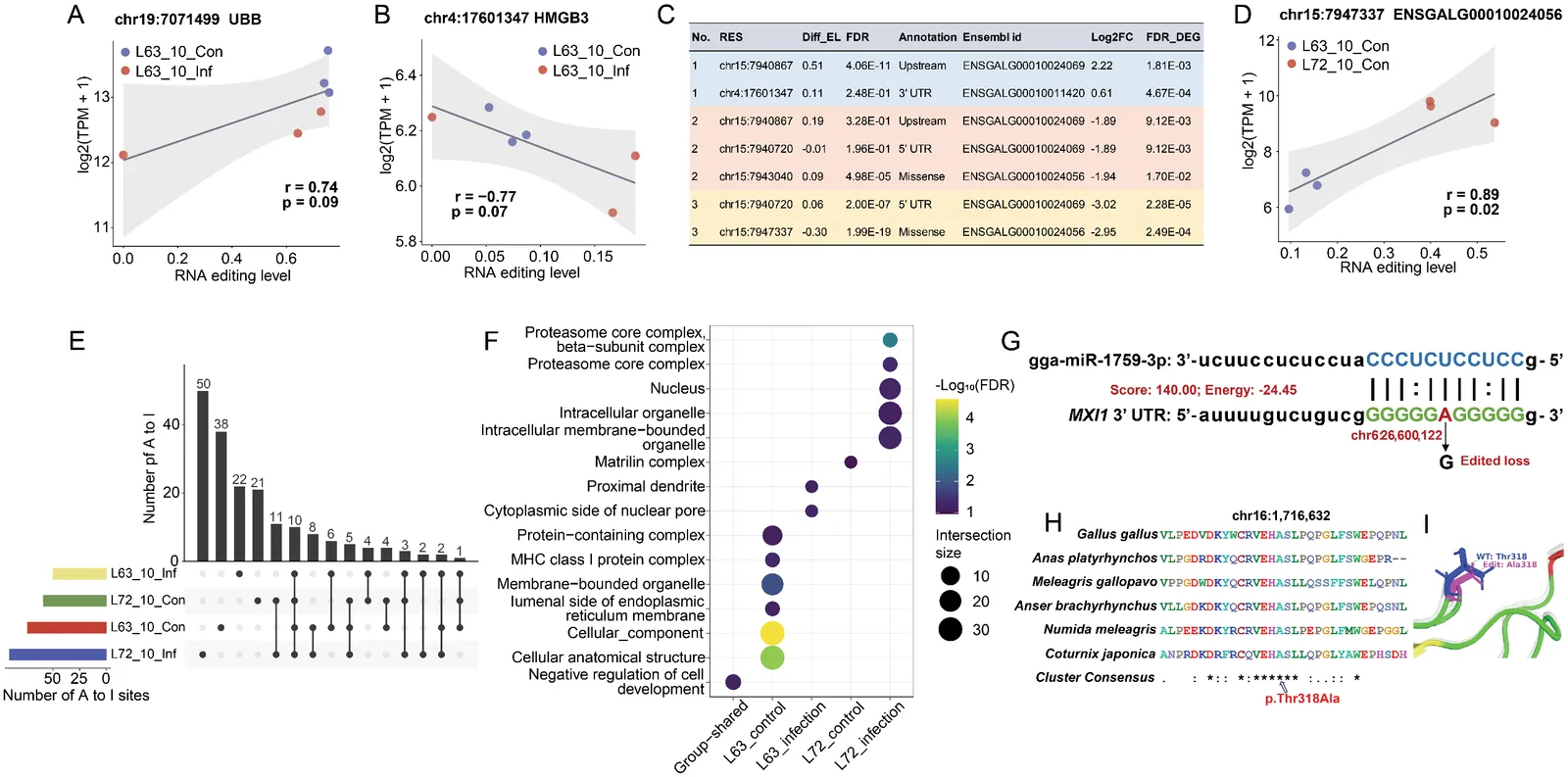
Comparative analysis of RNA editing identifies two candidate RESs associated with Marek’s disease. **A.** Editing level of differential RES (chr19:7,071,499) between infected and non-infected groups shows a positive correlation with LOC101747587 (*UBB*) expression. **B.** Editing level of differential RES (chr4:17,601,347) between infected and non-infected groups shows a negative correlation with *HMGB3* expression. **C.** Integration of differentially expressed genes and differential RNA editing sites across three comparisons. Each row represents a differential RES located within or near a DEG. Comparisons 1, 2, and 3 indicate L63-Inf versus L6_3_-Con, L7_2_-Inf versus L7_2_-Con, and L6_3_-Con versus L7_2_-Con, respectively. Diff_EL denotes the difference in editing level, FDR the adjusted significance of differential editing, Log2FC the change in gene expression, and FDR_DEG the adjusted significance of differential expression. Positive values of Diff_EL and Log2FC indicate higher RNA editing levels and gene expression, respectively, in the infected group for the within-line comparisons and in Line 63 for the between-line comparison. **D.** Editing level of differential RES (chr15:7,947,337) between resistant and susceptible chickens shows a positive correlation with ENSGALG00010024056 expression. The *P*-value and correlation coefficient were measured by Spearman's correlation test. **E.** UpSet plot shows the group-specific and group-shared A-to-I editing sites among four groups with different infection statuses in two chicken lines. **F.** The GO analyses were performed by g: Profiler for target genes of group-specific and group-shared RESs. **G.** Visualization of the gga-miR-1759-3p binding site in the *MXI1* 3’UTR. The RES at chr6:26,600,122 was predicted to alter the gga-miR-1759-3p binding site in the *MXI1* 3’ UTR. **H.** Multiple sequence alignment analysis for orthologous sequences of *MHCY57* across species. The RES at chr16:1,716,632 was predicted to result in a T318A substitution. **I.** Comparison of protein structure predicted by AlphaFold2 between before editing (wild) and after editing (edit).

In addition, RNA editing events across four groups were compared to identify group-specific editing sites, which may reflect distinct RNA editing patterns associated with MD resistance across the two chicken lines. We identified 38, 22, 21, and 50 group-specific RESs for L6_3_-control, L6_3_-infection, L7_2_-control, and L7_2_-infection, respectively. Meanwhile, 21 group-shared RESs were detected across at least 3 groups (Fig 4E, S4 Table). Functional enrichment analysis was performed for the corresponding genes revealed by group-specific RESs. The genes with L6_3_-control-specific RESs were significantly enriched in six cellular components, including MHC class I protein complex, protein-containing complex, etc., which may be related to MD resistance in the resistant chicken line. Genes identified by L6_3_-infection-specific RESs were enriched in the proximal dendrite and the cytoplasmic side of the nuclear pore. L7_2_-infection-specific RESs may be involved with the proteasome core complex. We also found that group-shared RESs show enrichment in negative regulation of cell development (Fig 4F). More importantly, we identified two candidate L6_3_-specific RNA editing sites (chr6:26,600,122 and chr16:1,716,632) that were significantly associated with *ADARB1* expression and the expression of their corresponding genes, *MXI1* and *MHCY57*. cDNA Sanger sequencing showed mixed transcript signals at both target positions, with estimated editing levels of approximately 42.3% for the *MXI1* site and 24.2% for the *MHCY57* site based on chromatogram peak heights (S4A, S4B Fig). These sites were predicted to be associated with changes in miRNA binding and amino acid sequences (S4 Table). RNA editing at chr6:26,600,122 within the *MXI1* 3’ UTR was predicted to weaken gga-miR-1759-3p binding, suggesting a potential effect on post-transcriptional regulation of *MXI1* (Fig 4G). Specifically, *MXI1* is a transcriptional antagonist of *MYC* that has been implicated in CD4⁺ T-cell differentiation and inflammatory regulation, including the modulation of Th17-associated GM-CSF and IL-17 expression [33–35]. The A-to-I RNA editing site at chr16:1,716,632 was predicted to result in a threonine-to-alanine substitution at residue 318 of *MHCY57*. Multiple sequence alignment analysis further suggested that this amino acid is more conserved after RNA editing across species (Fig 4H). Furthermore, this substitution at residue 318 was mapped to a predicted loop within the non-cytoplasmic Ig-like domain spanning residues 240–327 and may alter local hydrogen-bonding interactions and loop conformation (Fig 4I).

## Discussion

A-to-I RNA editing is an important post-transcriptional regulation mechanism that influences multiple biological processes and may modulate innate immune responses [15,36,37]. However, the relationship between A-to-I RNA editing and MD resistance in chickens remains largely unexplored. Here, we characterized the first systematic A-to-I RNA editing events in CD4⁺ T cells from chicken lines differing in MD resistance and MDV infection status to identify potential RNA editing sites associated with MD-resistance. We observed that most A-to-I editing events in CD4⁺ T cells are enriched in the 3’UTR region, consistent with previous reports [17,21,38]. Different from the enrichment of A-to-I RESs in Alu elements in mammals, we found that A-to-I RESs in chickens primarily reside in repetitive elements, particularly in the CR1 family [39]. Previous studies have suggested that RNA editing is associated with viral infection and may help limit viral infectivity in the host [27,40,41]. In this study, we observed a significant difference in the number of RESs between the infection and non-infection groups in two chicken lines. More importantly, we also found the divergence in RNA editing levels between the two chicken lines, with elevated RNA editing levels in the MD-resistant L6_3_ compared with the MD-susceptible L7_2_, which may reflect line-specific differences in host responses to MDV infection.

ADAR enzymes, including ADAR, ADARB1, and ADARB2, are crucial regulators of A-to-I RNA editing. Our results show that *ADAR* expression was higher than that of *ADARB1*, and *ADARB1* exhibited lower expression in infected samples. Previous studies have reported that ADAR expression is significantly associated with the number of RNA editing sites but not significantly correlated with RNA editing levels, similar to our findings [38,42]. Although we identified several RNA editing sites associated with *ADAR* expression in CD4^+^ cells, the majority of RESs showed no significant association with *ADAR* gene expression, suggesting that RNA editing is likely influenced by multiple regulatory factors such as RNA secondary structure [17]. In addition, our findings support the notion that A-to-I editing sites may be associated with multiple post-transcriptional processes, including alternative splicing, miRNA-target interactions, and protein recoding, contributing to transcriptomic diversity as reported across multiple organisms [37,43,44].

Our study further investigated differences in A-to-I RNA editing between infected and non-infected groups across chicken lines associated with MD-resistance. The differentially expressed gene ENSGALG00010024056 harbored two differentially edited RESs annotated as missense variants across the three comparisons. ENSGALG00010024056 was also reported as a differentially expressed and module-hub gene in the pituitary [45]. *BIRC2* and *SLAMF1* were captured at a splicing-related RNA editing site (chr1:182,466,974) and a 3’UTR RES (chr25:1,196,928), respectively, and exhibited higher RNA editing levels in the infected L7_2_. *BIRC2*, an inhibitor of apoptosis, was previously reported to be downregulated in both MD-resistant and MD-susceptible chicken lines and was also identified as a biomarker for duck hepatitis A virus type 1 infection [30,46]. Moreover, it was also reported to be associated with Kaposi’s sarcoma-associated herpesvirus infection and with immune cell ratios in femoral head necrosis in humans, suggesting a potential role in immune-related processes [47,48]. Extensive studies have reported that *SLAMF1* is involved in inflammatory and autoimmune diseases and is expressed in immunity-related cells such as activated B cells, T cells, and dendritic cells, which may support its potential role in host defense against MDV infection in chickens [49,50]. In our study, *HMGB3* harbored a 3’ UTR RES with an increased editing level in the infected L6_3_ group. Previous studies have implicated *HMGB* family proteins in nucleic acid-mediated innate immune sensing [32]. Qin et al. also reported that exosomal *HMGB3* was shown to promote inflammatory responses and monocyte/macrophage recruitment through activation of the STAT3, MAPK, NF-κB, and CCR2 pathways [51]. Moreover, *HMGB3* has also been implicated in innate immune responses to Tembusu virus infection in ducks [52]. Together, these findings suggest that *HMGB3* may be involved in host responses to MDV infection, although its specific role in CD4⁺ T cells remains to be determined.

Furthermore, our study also found that approximately 70% of RESs were group-specific; their corresponding genes were enriched in immunity-related GO terms, such as the MHC class I protein complex in Line 6_3_, consistent with previous findings in MD studies [53,54]. Our study identified a RES in the 3’ UTR of *MXI1* (chr6:26,600,122) that was predicted to alter gga-miR-1759-3p binding. The presence of this RES was associated with *ADARB1* expression and higher expression of *MXI1*, which encodes a transcriptional antagonist of MYC and has been reported to act as a tumor suppressor in multiple cancer types [35,55]. In avian infection studies, *MXI1* expression increased after infectious bursal disease virus infection, was linked to pathways associated with reduced lymphocyte abundance during Newcastle disease virus infection, and showed increased regulon activity after avian pathogenic *Escherichia coli* infection [56–58]. A previous study also reported gga-miR-1759-3p is associated with immune responses to H9N2 avian influenza virus infection [59]. Collectively, these findings suggest that the RES in *MXI1* may be relevant to host responses during MDV infection. *MHCY57* is located within the polymorphic MHCY region, the second major histocompatibility complex-like region in chickens. Previous studies have associated genetic variation in the MHCY region with susceptibility to MDV-induced tumors, the outcome of Rous sarcoma virus-induced tumors, differences in Campylobacter jejuni colonization, and variation in antibody responses [60–63]. In our study, the RES at chr16:1,716,632 resulted in a predicted threonine-to-alanine substitution at residue 318 of *MHCY57*. Multiple-sequence alignment showed that the edited alanine residue was more consistent with the conserved residue observed across species. These observations highlight this site as a candidate editing event, although its functional consequences on *MHCY57* during MDV infection require experimental validation.

Our study provides a systematic profile of A-to-I RNA editing in CD4⁺ T cells from MD-resistant and MD-susceptible chicken lines and identifies candidate editing events potentially associated with host responses to MDV infection. However, there are some limitations in our research, such as sample size, tissue class, and sequencing depth, and different time point after infection. In particular, because RNA editing was assessed only in splenic CD4^+^ T cells at 10 days post-infection, we cannot determine whether the observed editing patterns are protective or detrimental, or whether they represent a cause or consequence of differences in infection outcome. In addition, the low abundance of viral reads limited our ability to evaluate potential viral RNA editing events and their differences between the MD-resistant and MD-susceptible lines. Although distinct RNA editing patterns were observed between the two lines, these differences may also reflect variation in viral replication, inflammatory responses, or other infection-related processes. Future studies incorporating larger sample sizes, multiple tissues and immune cell types, deeper sequencing, and longitudinal sampling across different stages of infection will be required to characterize the temporal dynamics and functional significance of RNA editing. The integration of long-read sequencing with multi-omics approaches may further facilitate the construction of *ADAR*-associated regulatory networks and provide a more comprehensive understanding of the roles of A-to-I RNA editing in MD resistance and pathogenesis.

## Conclusion

In summary, our findings provide a systematic characterization of A-to-I RNA editing in CD4⁺ T cells during MDV infection and identify candidate RESs potentially associated with MD-resistance. These results provide a resource for future studies investigating the regulatory and functional roles of RNA editing in MD resistance.

## Methods

### Ethics statement

The animal study was approved by ADOL (31320-008-00D) and the University of Maryland (R-08-62). The study was conducted in accordance with the local legislation and institutional requirements.

### Experimental animals and chicken CD4^+^ T cell isolation

Two inbred lines of White Leghorn (Line 6_3_ and Line 7_2_) were hatched, raised, and maintained in the USDA-ARS Avian Disease and Oncology Laboratory (East Lansing, Michigan, United States). Chickens from each line were separated and divided into infected (treatment) and non-infected (control) groups (n = 3), and all chicks used in this experiment were maternal antibody-free for MDV. The vv + MDV strain 648A was used for viral challenge because previous studies have shown that this strain produces clear differences in MD incidence between MD-resistant Line 6_3_ and MD-susceptible Line 7_2_ chickens [64]. Chickens in the infected group were intra-abdominally challenged at 5 days post-hatch with the very virulent plus MDV strain 648A passage 10 at a dose of 500 plaque-forming units (PFU). The virus was propagated in duck embryo fibroblasts (DEFs). At 10 days post-infection, the chickens from the infection treatment and non-infection control groups were sacrificed following standard animal ethics and usage guidelines for tissue samples. The spleen tissue was temporarily stored in the medium (PBS with 2% FBS and 1 mM EDTA) for CD4^+^ T cell isolation preparation. Pellets were collected by centrifugation at 300g for 5 minutes. 5 mL of Ammonium-Chloride-Potassium (ACK) lysing buffer was added to lyse red blood cells for 5 minutes at room temperature. The cell pellets were recollected by centrifuging at 300g for 5 minutes. The cells were then resuspended to a concentration of 2 × 10^8^ cells/ml. 5% normal mouse serum (Cat. 0050-01, SouthernBiotech) was added to block non-specific antibody binding. 2µg of mouse anti-chicken CD4-PE antibody (Cat. No. 8210-09, SouthernBiotech) was added and mixed well. The binding was complete by incubating at room temperature for 15 minutes. Excess antibody was washed away by one wash with 10-fold PBS with 2% FBS and 1 mM EDTA. Cells were resuspended to the original volume for the EasySep PE Selection Cocktail from EasySep Positive Selection, PE Selection Kit (Cat. #18557, STEMCELL Technologies) was added at 100μl/ml cells and mixed well. The second binding was complete by incubating at room temperature for 15 minutes. EasySep Magnetic Nanoparticles from EasySep Positive Selection, PE Selection Kit (Cat. #18557, STEMCELL Technologies) were added at 50μl/ml cells and incubated at room temperature for 10 minutes. The splenic CD4^+^ T cells were enriched after three washes using a magnetic stand. After the last wash step, the tubes were removed from the magnet, and cells were resuspended in PBS with 2% FBS and 1 mM EDTA. The CD4^+^ T cell purity was assessed by flow cytometry, and an average 90%-95% purity was achieved. The CD4^+^ T cells were stored at -80°C.

### RNA preparation and sequencing

Approximately 50,000 isolated splenic CD4^+^ T cells were homogenized and stored in DNA/RNA Shield (Cat. # R1100, Zymo Research) for total RNA extraction. Total RNA from splenic CD4^+^ T cells in infected and uninfected groups was extracted using Quick-RNA MicroPrep Kit (Cat. # R1050, Zymo Research, USA) according to the manufacturer's instructions. cDNA was synthesized from polyadenylated RNA and amplified according to the Smart-seq2 protocol [65]. About 100ng of double-stranded cDNA was sonicated using the Bioruptor (Diagenode) to obtain random-sized DNA fragments. The dscDNA fragment ends were repaired by T4 and Klenow DNA polymerase, and the libraries were constructed using Illumina kits as previously described [65]. Each library was identified by adding a 6-bp adaptor and sequenced at 50-bp read by an Illumina HiSeq 3000 Sequencer [66].

### Data preprocessing and identification of A-to-I editing events

Raw single-end RNA-seq and paired-end whole-genome sequencing (WGS) reads from our previous study of these two chicken lines [67] were trimmed by fastp with default settings (v0.23.2) [68]. Clean reads were aligned to the reference GRCg7b genome using HISAT2 (v2.2.1) [69] and BWA-MEM (v0.7.17) [70] for RNA-seq and WGS. Aligned BAM files were sorted by Samtools (v1.15) [71], and MarkDuplicates in GATK (v4.6.2.0) was used to remove duplicated reads. REDItools3 was utilized to detect the A-to-I editing events based on deduplicated BAMs with the setting “-s 0 -q 25 -bq 30 -mbp 6 -Mbp 6” [72]. For DNA variants, we used the GATK tools FixMateInformation, BaseRecalibrator, and ApplyBQSR to ensure accurate mate-pair relationships and to reduce systematic sequencing errors through base quality score recalibration [73]. HaplotypeCaller was used for variant calling, and SelectVariants was applied to extract SNPs. The VCF files for six individuals were merged by VCFtools (v0.1.16) [74]. Finally, we obtained 6,726,261 SNPs.

To obtain the high confidence A-to-I editing sites, we applied the stringent filtrations, including (1) retaining sites supported by at least two edited reads and at least three total RNA-seq reads; (2) filtering sites overlapping with chicken SNPs published by EVA database (release 8) [75], and the SNPs detected above; (3) retaining sites identified in at least two individuals; (4) excluding sites with 100% editing level across all samples; (5) only considering A to G in positive-stranded genes and T to C in reverse-stranded genes as A-to-I editing.

### Functional annotation for A-to-I editing events

RNA editing sites were annotated using SnpEff v5.3 to characterize their genomic locations and predicted functional consequences [76]. All sites were annotated as 3’ UTR, 5’ UTR, intergenic region, intron, missense, upstream and downstream, and splicing-related etc. To annotate A-to-I RNA editing sites, the RepeatMasker annotation file corresponding to the chicken GRCg7b genome assembly was retrieved from the NCBI database. Gene Ontology (GO) and Kyoto Encyclopedia of Genes and Genomes (KEGG) enrichment analyses were conducted for RES-related genes using the g: Profiler2 tool [77].

To investigate the potential base preferences of RESs, we extracted the 10-bp upstream and downstream flanking sequences for each site. Moreover, RESs located within coding sequences (CDSs) were retained to extract the corresponding codons, and the resulting amino acid changes before and after A-to-I editing were compared using R packages GenomicRanges [78], BSgenome [79]. The proportion of base, codon, and amino acid at each position was visualized using ggseqlogo [80].

For RESs located in the 3’ UTR of the genes, miRanda v3.3a was used to predict miRNA binding with the unedited and edited 3’ UTR sequences with default settings [81]. All mature miRNA sequences for chicken were retrieved from miRbase. We extracted the flanking regions of the editing site (50 bp upstream and downstream) and generated edited and unedited sequences as candidate miRNA targets. We obtained candidate predicted miRNA-target interactions with energy less than -14 kcal/mol. Subsequently, candidate miRNA-targets were classified as edit gains or edit losses based on their presence in the reference and edited sequences.

### Quantification and comparative analysis of gene expression

Gene expression was quantified by StringTie (v3.0.1) based on aligned BAM files and genome annotation file (Ensembl, release-114) [82]. Raw read counts and TPM were extracted for studied samples, respectively. We only retained expressed genes with a read count of more than 10 in at least 3 samples for differential expression genes (DEG) analysis. DESeq2 was used to detect DEG for three comparisons including L6_3_-infection vs. L6_3_-control, L7_2_-infection vs. L7_2_-control, and L6_3_-control vs. L7_2_-control [83]. The threshold for identifying DEGs was set to false discovery rate (FDR) < 0.05 and an absolute log2 fold change (|log2FC|) > 1.5. TPM for ADAR family genes (*ADAR* and *ADARB1*) associated with A-to-I editing events were extracted to investigate their relationship with RNA editing. CD4⁺ T cells samples were then classified into edited and unedited groups for each editing site. A Wilcoxon rank-sum test was used to assess expression differences in *ADAR* and *ADARB1* between the two groups, enabling the identification of A-to-I editing sites whose presence was associated with *ADAR* or *ADARB1* expression. Similarly, associations between editing-site presence and expression of the corresponding genes were evaluated using the Wilcoxon rank-sum test by comparing expression levels between edited and non-edited groups [38].

### Identification of differential and group-specific RESs

Using Fisher’s exact test, differential RESs were identified for L6_3_-infection vs. L6_3_-control, L7_2_-infection vs. L7_2_-control, and L6_3_-control vs. L7_2_-control. Only RESs with a mean editing level greater than 0.01 in both groups were retained for differential analysis. Differential RESs were identified based on pooled RNA-read counts for each site (three replicates), including both edited and reference reads in the two comparison groups [18]. A site was considered a significantly differential RES if the FDR was <= 0.05 and the absolute difference in mean editing level between the two groups >= 0.01. Group-specific RESs were detected using UpSetR across the four groups [84]. RESs shared across groups were defined as those identified in at least three groups. Amino acid sequences of genes orthologous to chicken *MHCY57* were retrieved from goose, turkey, duck, and other species in Ensembl database [85]. Sequence conservation was assessed using ClustalW in the BioEdit software [86]. Protein structures of *MHCY57* before and after RNA editing were predicted with AlphaFold and visualized using PyMOL [87,88]. Protein domain boundaries were annotated according to UniProt, and membrane topology, including non-cytoplasmic, transmembrane, and cytoplasmic regions, was predicted using DeepTMHMM [89].

### Sanger sequencing for candidate RNA editing sites

To validate candidate RNA editing sites in *MXI1* (chr6:26,600,122) and *MHCY57* (chr16:1,716,632), total RNA from non-infected L6_3_ samples in which these sites were detected was reverse-transcribed using the AzuraFlex cDNA Synthesis Kit (AZ-1997). Regions spanning the candidate sites were amplified from cDNA using gene-specific primers (S8 Table) and NEBNext Ultra II Q5 Master Mix (M0544L). Amplicons of the expected sizes were excised from agarose gels, purified using the QIAEX II Gel Extraction Kit (Qiagen, Cat. No. 20021), and subjected to bidirectional Sanger sequencing with the corresponding forward and reverse primers by Molecular Cloning Laboratories (MCLAB) using an Applied Biosystems sequencing platform. The resulting .ab1 chromatograms were analyzed using sangerseqR, aligned to the corresponding GRCg7b reference sequences, and examined for nucleotide signals at the candidate editing positions.

## Supporting information

S1 Fig Read coverage distribution of identified RNA editing sites. **A.** Distribution of per-site read coverage across all identified RNA editing sites. The red dashed line indicates a coverage threshold of 10 reads. **B.** Distribution of read coverage for RNA editing sites in each sample. Boxplots are colored according to experimental group.
(TIF)

S2 Fig Associations between RNA editing and ADAR/ADARB1 expression. **A.** Density plot for editing level across four groups with different infected status in two chicken lines. **B.** Correlation between *ADARB1* expression and the number of RESs detected per sample. Each point represents one biological replicate. The solid line indicates linear regression, with the shaded area denoting the 95% confidence interval. **C.** Correlation between *ADARB1* expression and RNA editing levels. Editing level was calculated as the ratio of edited reads to total reads. **D.** Comparison of *ADAR* expression between samples with and without RNA editing. **E.** Comparison of *ADARB1* expression between samples with and without RNA editing.
(TIF)

S3 Fig Predicted functional consequences of A-to-I RNA editing. **A.** Predicted alteration of miRNA-target binding after A-to-I editing. **B.** Heatmap showing the RESs are associated with gene expression, and predicted regulatory consequences including alternative splicing, miRNA-target interactions, and amino acid alterations.
(TIF)

S4 Fig Sanger sequencing validation of candidate RNA editing sites in *MXI1* and *MHCY57.* Representative cDNA Sanger chromatograms are shown for *MXI1* chr6:26,600,122 (A) and MHCY57 chr16:1,716,632 (B) from non-infected Line 63 samples. The *MXI1* site is located on the forward strand and shows overlapping A/G signals, whereas the *MHCY57* site is located on the reverse-strand transcript and shows the corresponding T/C signals. Red dashed lines and arrowheads indicate the candidate editing positions. In the chromatograms, A, C, G, and T peaks are shown in green, blue, black, and red, respectively. The overlapping signals support the coexistence of edited and unedited transcripts. At the *MXI1* site, the mixed signal resulted in an ambiguous base call (“N”).
(TIF)

S1 Table High-confidence A-to-I RNA editing sites identified in chicken CD4⁺ T cells. (XLSX)

S2 Table A-to-I RNA editing sites associated with *ADAR* and *ADARB1* expression. (XLSX)

S3 Table Associations between A-to-I RNA editing sites and the expression of their corresponding genes. (XLSX)

S4 Table Functional annotations and predicted consequences of A-to-I RNA editing sites. (XLSX)

S5 Table Predicted changes in miRNA-target interactions associated with A-to-I RNA editing. (XLSX)

S6 Table Differentially expressed genes identified across three comparisons involving chicken line and infection status. (XLSX)

S7 Table Differential A-to-I RNA editing sites identified across three comparisons involving chicken line and infection status. (XLSX)

S8 Table Primer sequences used for Sanger sequencing validation of candidate RNA editing sites. (XLSX)

S1 Source data Gene expression data from RNA-seq samples of Marek’s disease-resistant Line 6_3_ and susceptible Line 7_2_ chickens under MDV-infected and non-infected control conditions. The dataset includes gene expression levels expressed as raw read counts and transcripts per million (TPM) and for 12 samples, with three biological replicates per group.
(XLSX)
